# Long range correlations of stride intervals in uphill and downhill trail running

**DOI:** 10.3389/fspor.2025.1679343

**Published:** 2025-11-14

**Authors:** Matteo Genitrini, Jon Wheat, Hermann Schwameder

**Affiliations:** 1 Institute of Sport Science and Innovations, Lithuanian Sports University, Kaunas, Lithuania; 2 Department of Sport and Exercise Science, University of Salzburg, Hallein-Rif, Austria; 3 Sport and Human Performance Enhancement Research Centre, Nottingham Trent University, Nottingham, United Kingdom

**Keywords:** trail running, DFA, uphill, downhill, motor control

## Abstract

**Introduction:**

Trail running is an increasing popular endurance discipline. The goal of the present study was to investigate long range correlations in stride intervals during a full trail running time trial.

**Methods:**

Adopting an exploratory approach, it was hypothesized that the strength of such correlations would differ between uphill and downhill sections and between the initial and final stage of the race (incline and stage as independent variables). Twenty participants were recruited to run a solo all-out time trial equipped with inertial sensors to calculate stride intervals. The strength of long range correlations in stride intervals was quantified by means of Detrended Fluctuations Analysis alpha exponents (DFA-alpha). Differences across conditions were tested by means of linear mixed effect models.

**Results and discussion:**

A significant main effect for incline was found, with higher values of DFA-alpha in downhill sections (resulting from less tight control) with respect to uphill. This is likely due to the higher technical difficulty running at high speed on an uneven surface. A significant main effect was found for race stage, with stronger correlations in the second race half as compared to the first one, most likely resulting from the difficulty to regulate running cadence in presence of acute fatigue. A significant interaction between incline and race stage was found as well, indicating that the strength of long range correlations in the second half of the race increased in both uphill and downhill sections, but the increase was significantly larger in uphill sections. This is likely due to the increase in physical fatigue which is prevalent in uphill sections, whilst the technical difficulty of downhill section remains constant. The present study shows that DFA-alpha is a sensitive quantity to discriminate between more and less challenging motor control scenarios. Incorporating such DFA-alpha among the metrics provided by wearables may aid runners in choosing a pacing strategy aiming to minimize fall and injury risks.

## 1 Introduction

Trail running is an increasingly popular endurance discipline, defined as any foot race taking place in a natural environment (e.g., mountains, forests, deserts etc.). Not more than 20–25% of the race length can be paved or asphalted, with athletes spending most of the time on a trail, dirt road or a single track; there are no limits to the race length and elevation gain, with race distance ranging from ∼five to several hundred kilometers (1). One of the most demanding factors in trail running is the incline, with the potential for long and technically challenging uphill (UH) and downhill (DH) sections.

Previous works have highlighted how the movements patterns of trail runners differ depending on performance standard of the runner, as well as on the stage of the race and the gradient of the terrain (2, 3). Faster athletes appear to have lower energy absorption and more favorable net mechanical work at the knee joint in UH sections. In DH sections faster athletes show a more efficient motion of the swing leg (higher hip and knee peak flexion angles), which serves to increase momentum in the forward direction and full body center of mass’ velocity at toe off, thus optimizing the propulsion phase of the contralateral leg (2). With respect to the effect of acute fatigue in late race stages, athletes have been reported to express lower energy generation at the ankle joint in UH sections, whilst changes in the kinematics of swing leg in DH sections may contribute to reducing the effectiveness of the propulsion phase (3).

In recent years, alongside traditional kinematics and kinetics, nonlinear analysis has gained increasing attention in sports as a valuable tool to provide insights about motor control and temporal organization of time series derived from biological processes. In particular, detrended fluctuation analysis (DFA) represents a valuable tool to investigate variability of key gait parameters, such as stride intervals. Whilst quantities such as standard deviation indicate the magnitude of variability, they do not provide information about its temporal organization. Conversely, DFA quantifies long range correlations in stride intervals, whose distribution has been ascertained to be a fractal process (4, 5). This means that a given stride interval is dependent on previous stride intervals at a remote time and that the dependence of stride intervals decays in a power law, fractal-like manner with time (4–6). Power law decay means that those processes which exhibit long range correlations are characterized by 1/f-like frequency content, with large fluctuations occurring at low frequency and small fluctuations occurring at high frequency. Moreover, fractal-like biological processes are characterized by self-similarity, “i.e., small irregularities at small time scales have the same statistical properties as large irregularities at larger time scales” (6). Fluctuations exhibit either persistence or anti-persistence. Persistence means that a long stride interval is likely followed by another long interval, and vice versa. Anti-persistence indicates that a long stride interval is likely followed by short interval, and vice versa.

Several investigations have quantified long range correlations in stride intervals during running. Meardon et al. (7) reported that acute fatigue decreases persistence in stride intervals, i.e., more frequent corrections were needed to run at constant speed on a an indoor track, as exercise time increases . However, Mo and Chow (8) reported the opposite, with higher persistence in the final stages of a treadmill run. As such, the effect of exercise time on stride interval long range correlations in running is unclear. The effect of terrain slope has also been investigated. During an outdoor parkrun, higher persistence of stride intervals has been reported in UH running, with respect to DH (9). To our knowledge, non-linear analysis of stride intervals has not been reported in trail running. Information about temporal organization of stride intervals would provide insights about how the motor system regulates running cadence on different inclines (UH vs. DH), as well as in different race stages (initial vs. final). Such findings would enhance current understanding of the challenge to the motor system associated with different constraints in trail running, e.g., fatigue or running surface, positive or negative incline. In turn, this would enable athletes and coaches to specifically emphasize preparation for those sections of the trail running route that present the greatest challenges in this regard. Therefore, the aim of this study was to investigate long range correlations in stride intervals during a full trail running time trial, comparing UH vs. DH and the initial vs. final stages, as well as the interaction of incline and race stage.

## 2 Materials and methods

### 2.1 Participants

Twenty participants (10 M, 10 F) were recruited from local trail running clubs (age [years]: 32.8±8.3 M, 33.4±8.1 F; stature [cm]: 177.2±6.0 M, 166.3±6.9 F; body mass [kg]: 71.9±5.8 M, 61.6±6.9 F, experience in trail running [years]: 3.3±1.5 M, 4.1±1.2 F). All participants were amateur athletes who regularly competed regionally or nationally. The study was approved by the Ethics Committee , in accordance with the Declaration of Helsinki.

Participants were included if they were being 18–50 years old, had at least 1 year of trail running experience, trained at least twice and ran at least 30 km per week and had no injuries for at least three months before the study.

### 2.2 Protocol

**Figure 1:**
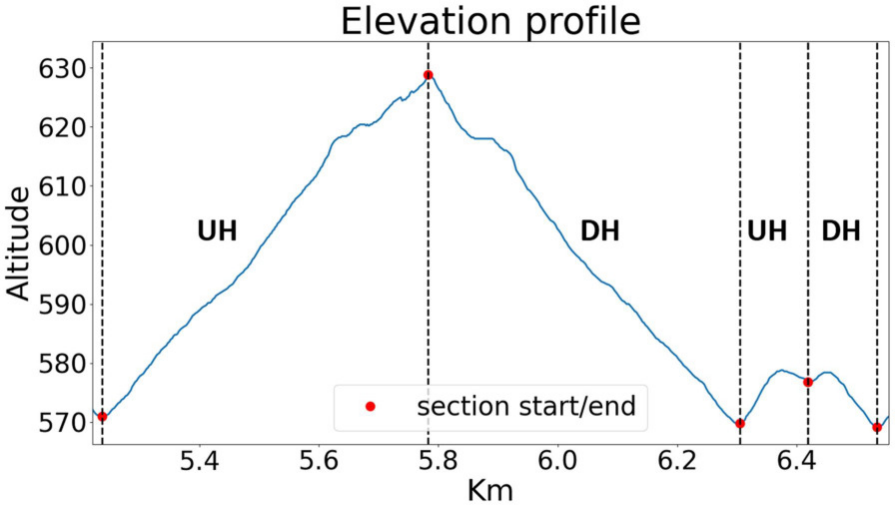
Elevation profile of a trail running time trial lap. Data are from a typical lap of a typical participant; a full test consisted of seven repetitions.

Participants were required to attend two testing sessions on separate days. On a first day, participants provided informed consent. Subsequently, anthropometric data were recorded, including stature and body mass. On a second day, participants completed a ∼9.1 km trail running time trial consisting of 7 laps of the same 1.3 km route (see Figure 1). Laps 1-3 were classified as first race stage, whilst laps 5–7 were classified as second race stage. Each lap presented an elevation gain of 60 m, resulting in 420 m of elevation across the entire trial. Before the time trial, participants were accompanied during a complete lap of the running route to familiarize themselves with the test environment, followed by a self-selected warm up, consisting of level and incline running, as well as static and dynamic stretching exercises. Participants were instructed to complete the test in the shortest time possible, without jeopardizing their safety.

**Figure 2:**
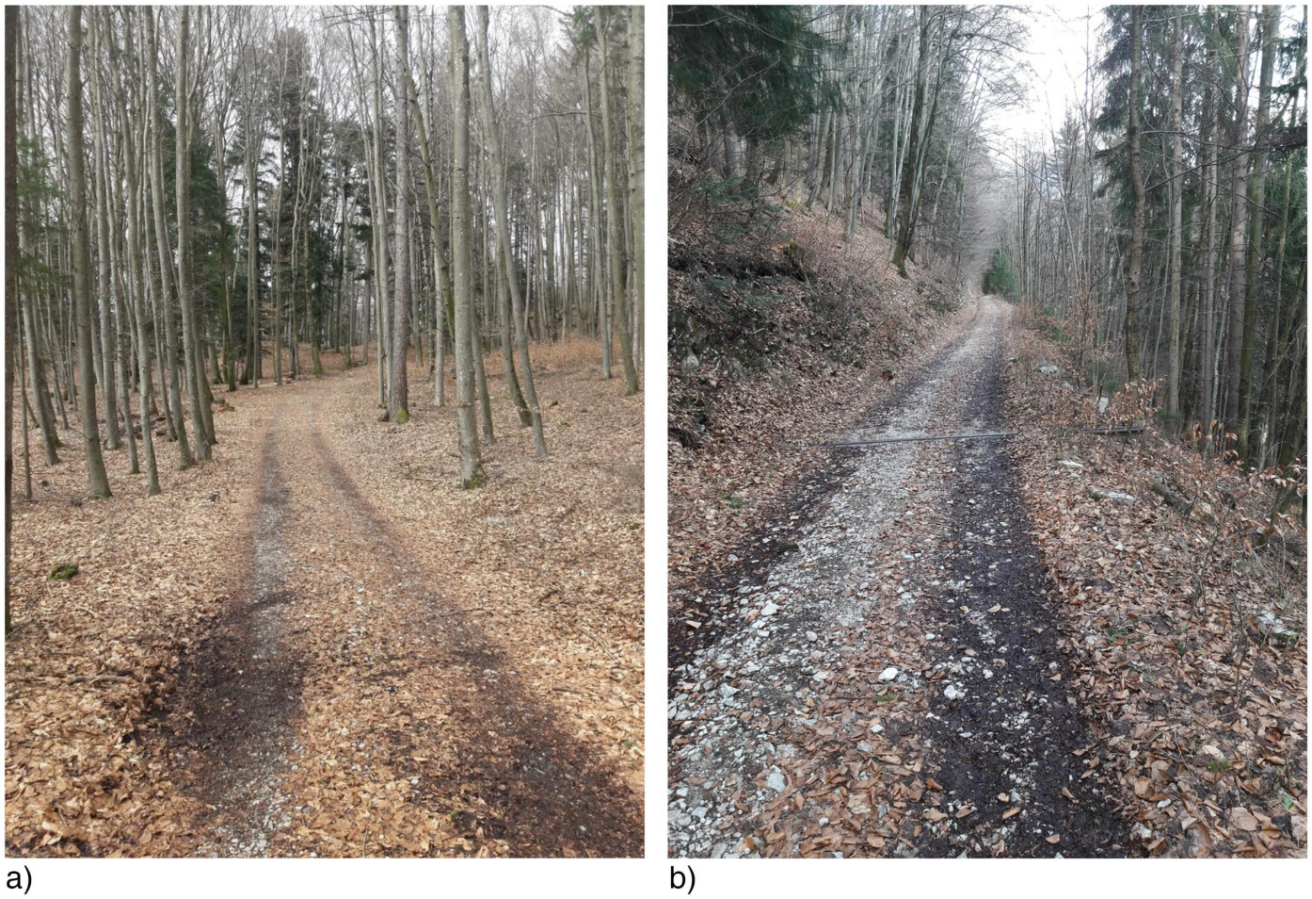
Ground morphology at two different locations of the trail running route **(a,b)**. Terrain was a combination of soil and gross grain gravel, with a thin layer of pine needles and leaves on top. This combination made the ground uneven but yet safe to run. Also, it presented good water absorption, making it stable in case of tests on the day after rain.

All participants were tested between April–August. The temperature during tests was 23.2 ± 3.7∘C, with sunny or cloudy weather conditions. No tests were performed in the rain. Within each lap, two UH and two DH sections were considered (see Figure 1). The first UH and DH sections were on a trail, whilst the second UH and DH sections were on asphalt. Ground morphology consisted of soil and uniform gross grain gravel (stones of ∼ 5–7 cm), with a thin layer of pine needles and leaves on top (Figure 2). This combination gave the ground good stability and water absorption characteristics, making the route weather-proof and safe to run also in case of rain on the day before testing. In the present study, data from the trail part only were considered.

### 2.3 Materials

Participants wore a GPS watch (Garmin Forerunner 935) and a full-body motion capture system (Xsens Link, Xsens Technologies BV, Enschede, The Netherlands), consisting of 15 inertial measurement units (IMUs, model MTx, size 36×24.5×10 mm, mass 10 g, sampling frequency 240 Hz). IMU sensors were located on the head, shoulders (2×), arms (2×), forearms (2×), thighs (2×), legs (2×), feet (2×), sternum, and pelvis. Specifically, the athletes wore a tight lycra suit, and sensors were attached to velcro patches embedded in the suit, in turn located into a skin-tight zip pocket to ensure stability. Previous studies validated this system against gold standard marker-based methods, reporting reliable and consistent results for tasks such as running and changes of directions on both asphalt and uneven surfaces (10–13). All participants wore the equipment from approximately 30 min before the beginning of the time trial, thus having enough time to become familiar with the suit and the wearables. Those participants who asked for it, were additionally allowed to run with their own watch, meaning they wore one at each wrist.

### 2.4 Walking gait cycles

Running is characterized by a flight phase where neither foot is in contact with the ground. The ratio of ground contact time to stride time is referred to as duty factor (DF). Values lower than 50% indicate running, whilst values above such threshold indicate walking.

In trail running it is not unusual to switch between running and fast walking, especially in UH sections. Nonetheless, since the present study focuses on running biomechanics, participants walking more than 10% (DF less than 50%) of total gait cycles were excluded. Participant did not walk in DH sections.

### 2.5 Stride intervals and long range correlations

By means of a previously validated algorithm (14), linear acceleration peaks of the foot dorsum sensor were used to identify initial contact and toe off. Stride intervals were defined as the time elapsed between two consecutive ipsilateral initial contacts.

Long range correlations were assessed using equally spaced detrended fluctuation analysis (esDFA) (15). Conceptually, it is assumed that the standard deviation of the integrated series is a power function of the interval length over which it is computed, with an exponent alpha, hereafter referred to as DFA-alpha. The algorithm works as follows. The time series is first integrated, and then it is divided into n non overlapping windows of length l. Within each interval the series is detrended, and the standard deviation of the residuals is computed. Then, the average standard deviation across all n intervals of length l is calculated. This value forms one data point on the log-log plot, showing the value of l on the x-axis and the average standard deviation on the y-axis, both expressed on logarithmic scale. Subsequently, the process is repeated across different values of l. When using esDFA, a number k of different interval lengths for l are chosen so that the k different values of l are evenly spaced on the horizontal axis of the log-log plot. Finally, DFA-alpha is computed as the slope of the linear regression of all the k data points previously obtained. In the present work, as an input to the algorithm, interval lengths ranging from 4 to L/2 were used, where L is the length of the time series, comparably to previous works (6). A number k=10 equally spaced interval lengths was used. Since stride length is longer in DH sections, fewer stride intervals were available, compared to UH sections where stride length is shorter. To overcome this potential source of bias, only the central M stride intervals were used for UH sections, where M is the number of stride intervals in the corresponding DH section. Values of DFA-alpha close to 1 indicate persistence in long range correlations between stride intervals in the time series (i.e., after a long stride interval, another long stride interval is likely to occur); values of DFA-alpha close to 0.5 indicate randomness, also referred to as *white noise*; finally, values of DFA-alpha close to 0 indicate antipersistence in long range correlations (i.e., after a long stride interval, a short stride interval is likely to occur).

In addition to DFA-alpha, standard deviation of stride intervals and running speed were computed, in order to provide information relative to the absolute magnitude of variability and overall performance, alongside its temporal organization of stride intervals.

### 2.6 Data reduction and statistics

Six participants were removed from the analysis due to excessive walking i.e., more than 10% of total gait cycles, resulting in a sample size of 14 individuals (6 F, 8 M).

With respect to magnitude of stride interval variability, i.e., standard deviation, possible effects of incline and race half were tested by means of a linear mixed effects model (LMEM). A LMEM was implemented with the standard deviation of stride intervals as the dependent variable. Race half, incline and their interaction were included as fixed effects, with Participant ID (intercept) also included as a random effect. Both race half and incline were treated as categorical variables and contrast-coded.

With respect to the temporal organization of stride intervals, prior to further analysis, a surrogation technique was used to statistically distinguish between actual long range correlations and random processes as in previous works (4). In particular, for each time series (i.e., for each participant in each lap for both UH and DH sections) 20 surrogate time series were produced by random shuffling the original data. Surrogate time series had the same mean and standard deviation as the original data, and differed only for the temporal sequencing of the data points. The mean DFA-alpha across the 20 surrogate time series was computed, as well as its standard deviation. If the difference between the DFA-alpha of the original time series and the mean DFA-alpha of the 20 surrogate time series was larger than 2 standard deviations, the long range correlations were considered not to be due to chance. Subsequently, a LMEM was implemented. DFA-alpha was the dependent variable. Fixed and random effects were identical to the model for the standard deviation of stride intervals described above.

**Figure 3:**
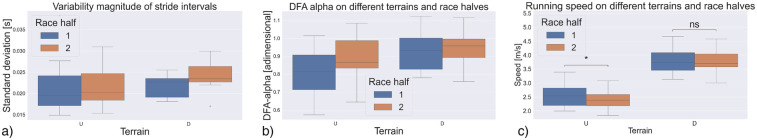
(**a**) data distribution of variability magnitude of stride intervals, i.e., standard deviation. (**b**) data distribution of temporal organization of stride intervals, i.e., DFA-alpha. (**c**) data distribution of running speed across conditions. U = Uphill, D = Downhill, Ns = non significant.

The quality of the models (for both magnitude and temporal organization of stride intervals) was assessed by visually inspecting the QQ plots and the distribution of the residuals. With respect to running speed, separately for UH and DH sections, difference between race halves was tested via paired t-test. The influence of incline was not investigated as it is apparent that speed is higher in DH running (see Figure 3c).

## 3 Results

### 3.1 Magnitude of stride intervals variability

A significant main effect of incline was found (Table 1), with lower values in UH sections compared to DH (−0.0015 s, i.e., 6.7%, Figure 3a). Conversely, no main effect of race half or a significant interaction were observed, indicating that the magnitude of variability was not affected by race stage and that the difference between UH and DH was similar, during the first and second half of the race.

**Table 1:** Linear mixed effect model output for the standard deviation of stride intervals.

Predictors	Estimates	CI	p
Intercept	0.0214	0.0199–0.0230	**<0.001**
Incline	−0.0015	−0.0029 to −0.0000	**0.043**
Race half	0.0011	−0.0003 to −0.0026	0.125
Incline * race half	−0.0018	−0.0046 to 0.0011	0.225

CI, confidence interval; *p*, *p*-value.

Bold values represent the statistically meaningful effects.

### 3.2 Temporal organization of stride intervals variability

The results relative to the surrogation test are reported in the Supplementary Material. With respect to the full dataset, results are presented in Tables 1, 2.

**Table 2:** Linear mixed effect model output for the DFA-alpha of stride intervals.

Predictors	Estimates	CI	p
Intercept	0.89	0.84–0.94	**<0.001**
Incline	−0.08	−0.11 to −0.05	**<0.001**
Race half	0.05	−0.01 to −0.08	**0.006**
Incline * race half	0.07	0.01–0.14	**0.031**

CI, confidence interval; *p*, *p*-value.

Bold values represent the statistically meaningful effects.

DFA-alpha was significantly different between UH and DH running, with lower values in UH sections (−0.08, i.e., −8.5%, Figure 3b). There was also a significant main effect of race half, with larger values of DFA-alpha in the second race half (+0.05, i.e., +5.4%). Further, there was a significant interaction, indicating that the difference in DFA-alpha between race halves is significantly larger in UH sections.

### 3.3 Running speed

Running speed was significantly lower in the second race half in UH sections (p<0.05). With respect to DH sections instead, no difference was found between race halves.

## 4 Discussion

The aim of this study was to investigate the effect of incline and race stage on the variability magnitude and long range correlation of stride intervals during a trail running time trial. Stride intervals standard deviation was lower in UH sections (−0.0015 s, i.e., −6.7%). Further, UH running was associated with lower DFA-alpha values than DH running (−0.08, i.e., −8.5%). DFA-alpha values were also significantly lower in the second race half (+0.05, i.e., +5.4%). Finally, a significant interaction between incline and race stage showed that the difference in DFA-alpha between race halves was greater in UH than in DH sections.

The larger standard deviation of stride intervals in DH sections (−0.0015 s, i.e., 6.7%) suggests that participants needed to make continuous small adjustments while running at high speed on an uneven ground. This was especially evident on DH inclines, where the running speed is greater and the reaction time to avoid stones and obstacles is shorter than UH running. During UH running, the time to collision with any potential obstacles is larger, and athletes do not need to make small time scale alterations immediately before landing, resulting in a more regular gait in terms of stride intervals.

It has been suggested that anti-persistence (i.e., DFA-alpha in the range 0–0.5) indicates tighter motor control, as a longer stride interval is promptly followed (i.e., corrected) by a shorter one (16). Depending on the constraints (i.e., the boundary conditions that combine to shape performance), this could be positively interpreted as an indicator of adaptability or, more negatively, as an indicator of over rigidity. Persistence (i.e., DFA-alpha in the range 0.5–1), on the other hand, may be interpreted as an indicator of less stringent motor control (7, 16). Overall, long range correlations of stride intervals should be interpreted carefully by holistically considering the ensemble of boundary conditions constraining performance.

Zignoli and colleagues reported larger values of DFA-alpha (stronger persistence) when running at higher speeds, as well as when running UH as compared to DH (17). When analyzing long range correlations during an overground mass-start event (a parkrun) with UH and DH sections presenting a ∼2% slope, Jones and colleagues reported larger values of the DFA-alpha in UH (9). These results contradict the findings of the present study. To explain this apparent conflict, we consider the ensemble of constraints that participants experienced during the testing procedure. In both the aforementioned studies, the running surface was smooth and did not present any technical difficulty (a treadmill in Zignoli et al. and asphalt in Jones et al.). Furthermore, in both studies, the gradient was shallower than the 10–12% of the present investigation. Due to the work required to move the center of mass against gravity, running UH is inherently more energetically challenging than running DH. Consequently, environmental constraints are more demanding when running UH, than running DH. Increased constraint has been associated with higher biological stress (6, 18). We suggest that running UH on a smooth surface such as a treadmill (17) or asphalt (9) where all the effort is physical and not technical may induce biological stress that lowers the amount of control over stride intervals, resulting in higher DFA-alpha in UH slopes. Further, Jones et al. (9) investigated the temporal organization of stride intervals during overground running in a mass-start event. We suggest that the lower DFA-alpha reported in this work in DH sections (less persistent or, equivalently, more antipersistent) might have also resulted from the tighter control that participants had to exert to avoid other runners, for instance accelerating and decelerating depending on the clusters of athletes. A scenario like this would require more stringent control of stride intervals compared to UH sections, where collisions with other athletes are less likely due to lower running speed, which, in turn, would allow for less tight control over temporal organization of stride intervals. Contrary to these investigations, data presented in this study were collected on a unpaved surface, consisting of soil, gross grain gravel and pine needles, with an average slope of ∼11% (see Figure 2). When running UH, the main challenge is physical, minimal impact from the irregularity of the terrain due to the lower running speed. The situation is reversed when running DH, characterized by lower physiological demand and higher technical difficulty arising from running at high speed on an irregular surface. One of the key findings of this study is the higher DFA-alpha in DH sections (+0.08, i.e., +8.5%). This suggests that, in terms of motor behaviour, the technical demands of DH running, caused by higher running speed on uneven ground) pose a greater challenge and induce more biological stress than the physiological demands of UH running. When running DH at high speed on uneven terrain, continuous small corrections and adjustments were needed. This increased both the magnitude of variability in stride intervals (i.e., standard deviation) and persistence in their temporal organization, since the priority was to maintain balance and avoid obstacles, rather than tightly regulate stride intervals around a fixed value. Interestingly, it might be expected that small corrections would lead to a long stride interval being followed by a shorter one and vice versa, i.e., anti-persistence and values of DFA-alpha below 0.5. Since the values of DFA-alpha are larger than 0.5 (indicating that a long stride interval is followed by another long stride interval and vice versa), the present data show that athletes “correct” the temporal organization of their strides so to minimize the difference between two consecutive stride intervals. Indeed, to avoid abrupt changes in running speed (i.e., performance), stride length should vary consistently to stride intervals, namely long stride intervals combined with long strides, and vice versa. Therefore, the present data suggest that athletes seek to minimize changes in the motor strategy used to run at a certain speed, since abrupt variations would be detrimental form an energetic standpoint and would negatively impact performance.

There was a significant effect of race half on DFA-alpha, with greater persistence in stride intervals in the second race half (+0.05, i.e., +5.4%) than the first. This supports the idea that biological stress - such as fatigue in the later stages of the race -reduces flexibility and adaptability, thereby strengthening long-range correlations in stride intervals (6, 19). At the same time, running speed in UH sections significantly decreased in the second half, while DH sections showed no change (Figure 3c). This suggests that race half had a greater impact on performance in UH sections. Indeed, a significant interaction between race half and incline indicates that DFA-alpha values were increased in the second half of the time trial in both UH and DH sections, but the increase was significantly larger in the UH sections. This suggests that biological stress related to physiological demand and fatigue in the UH sections increased over the course of the race, leading to stronger persistence in the temporal organization of stride intervals. In contrast, the dominant constraint in the DH sections - technical difficulty - did not change between race halves, resulting in less of a change in DFA alpha values. This interpretation is consistent with studies on expert runners that have shown an increase in stride intervals DFA-alpha, as exercise time increases (8). Furthermore, Montull et al. (20) recently reported an increase in the persistence of upper back acceleration in the final stages of an uphill trail running time trial. Moreover, previous works addressed how fatigue itself, and not incline, influence long range correlations during level running on a treadmill (21). The authors reported increased persistence as for the knee joint with higher levels of fatigue.

This is the first study to examine the temporal organization of stride intervals during a trail time running time trial in ecological conditions. Overall, results indicate that the technical difficulty of DH sections represents a more stringent constraint than the physiological demands] in UH sections, with higher DFA-alpha in DH suggesting a less tight motor control due to the increased difficulty associated to making small adjustment while running at high speed on an uneven surface. Further, stride intervals became more persistent in the second race half, reflecting the greater biological stress induced by increased physiological demands and fatigue as the race progressed. The difference between race stages, however, was significantly larger in UH sections, i.e., where physical fatigue increased the most dominant. A smaller difference was seen between race halves in the DH sections, in which the important constraints related to technical difficulty did not change as the race progressed.

This study also adds to the body of evidence that DFA-alpha can be more sensitive than traditional variability metrics (such as standard deviation) in discriminating between more and less challenging motor behaviour scenarios. Since the algorithm is simple to implement, we suggest that incorporating 308,292 DFA alpha into wearables such as smart watches could provide valuable information for the athletes. Changes in DFA-alpha reveal changes in temporal organization stride interval due to changes in physiological demand and fatigue when traditional metrics such as running speed and cadence have not changed. More awareness in this regard could ultimately lead to more cautious pacing strategies, for example, which could in turn result in lower injury rates within trail running community.

## Data Availability

The raw data supporting the conclusions of this article will be made available by the authors, without undue reservation.
