# Supplementary material for: Long range correlations of stride intervals in uphill and downhill trail running
Source: Front Sports Act Living. 2025 Nov 14;7:1679343. doi: 10.3389/fspor.2025.1679343 (PMC12660192; doi:10.3389/fspor.2025.1679343)
Supplement: Supplementary file 1 [file Datasheet1.pdf]

Supplementary Material

1 SUPPLEMENTARY DATA

In this section we report the results relative to the surrogation test described in the Materials and Method section of the main body.
Surrogation revealed that 20.5% of alpha exponents in the original data were due to chance (37/181 data points). The results relative only to the data that passed the surrogation test (i.e. the remaining 79.5% of the data points) were tested similarly to the full dataset presented in the main body of the present work. A LMEM was implemented. Alpha exponent was the dependent variable in both models. Race half and terrain were the fixed effects. Their interaction was tested as well. Random effect were the individual participants. Race half and terrain were set as categorical variables and contrast-coded. In particular, the reference category for race half was the first half and the reference category for terrain was downhill. The quality of the models (for both mmagnitude and temporal organization of stride intervals) was assessed by visually inspecting the QQ plots and the distribution of the residuals.
The summary of the model is reported in figure S1.From a qualitative standpoint, results were identical to the model run on the full data set, which corroborates the reliability of the present findings. In particular, alpha exponents significantly differed on different terrains, with lower values in UH sections (-0.05, i.e. -5.6%). Also, a significant main effect of race half was present, with larger values of alpha exponents in the second race half (+0.03, i.e. 3.8%). Also, a significant interaction was found, indicating that the difference of alpha exponents between race halves is significantly larger in UH sections.

		**alpha**	
Predictors	*Estimates*	*CI*	*p*
(Intercept)	0.93	0.89 – 0.96	**<0.001**
terrain	-0.05	-0.08 – -0.02	**0.001**
rh	0.03	0.00 – 0.07	**0.027**
terrain × rh	0.06	0.00 – 0.12	**0.040**

Figure S1. *Results of LMEM run on DFA-alpha of stride intervals. Only the strides which passed the surrogation test were retained. Predictors are the main effects (terrain UH or DH, race half 1st or 2nd, and their interaction). 95% confidence intervals and p-values are reported for each main effect as well.*
